# Well-defined BiOCl colloidal ultrathin nanosheets: synthesis, characterization, and application in photocatalytic aerobic oxidation of secondary amines†
†Electronic supplementary information (ESI) available: Experimental details, XRD patterns, TEM and HRTEM images, energy-dispersive X-ray spectra, UV-vis spectra, and Tauc plots. See DOI: 10.1039/c4sc03229b



**DOI:** 10.1039/c4sc03229b

**Published:** 2014-12-22

**Authors:** Yihui Wu, Bo Yuan, Mingrun Li, Wen-Hua Zhang, Yan Liu, Can Li

**Affiliations:** a State Key Laboratory of Catalysis , Dalian Institute of Chemical Physics , Chinese Academy of Sciences , Dalian National Laboratory for Clean Energy , Dalian 116023 , China . Email: whzhang@dicp.ac.cn ; Email: yanliu503@dicp.ac.cn ; Email: canli@dicp.ac.cn

## Abstract

We demonstrate the first colloidal synthesis of single-crystalline BiOCl ultrathin nanosheets (UTNSs) that feature a well-defined square morphology. Unlike BiOCl nanomaterials prepared by hydrothermal routes, our colloidal BiOCl UTNSs exhibit hydrophobic surface properties and high activity and selectivity toward the photocatalytic aerobic oxidation of secondary amines to corresponding imines at room temperature. Hence, the application of BiOCl nanomaterials has been successfully extended from the widely studied photodecomposition of pollutants in aqueous solution to the synthesis of fine chemicals in organic solvent using a green approach.

## Abstract 2

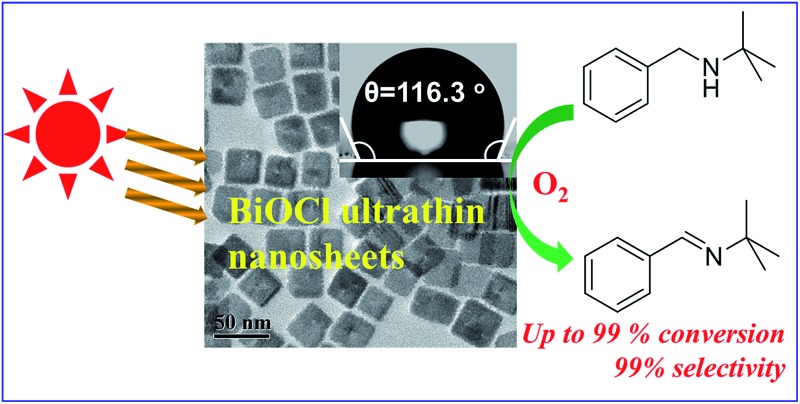
Well-defined BiOCl ultrathin nanosheets were prepared by a facile colloidal route, and exhibit high photocatalytic performance toward the oxidation of secondary amines to corresponding imines under visible irradiation.

## Introduction

Imines are important synthetic intermediates involved in the preparation of pharmaceuticals and biologically active nitrogen-containing organic compounds.^1^ Their broad application has generated considerable research interest in developing synthesis routes toward their fabrication.^2^ Direct oxidation of amines to corresponding imines using molecular oxygen is an ideal green route. Consequently, several catalytic systems have been explored, but only few amines can be successfully oxidized to imines using expensive metals at relatively high temperatures.^3^ Recently, considerable efforts have been made toward using inorganic semiconductors, as alternatives, for the synthesis of imines.^4^ As exemplified, mpg-C_3_N_4_,^4*c*^ TiO_2_,^4*d*^ Nb_2_O_5_,^4*e*^ Au/TiO_2_,^4*f*^ CdS,^4*g*^ and LDH^4*h*^ have been successfully used to prepare imines *via* the oxidation of amines. However, the use of inorganic semiconductors as photocatalytic systems is mainly limited to the oxidation of primary amines. In contrast, the aerobic oxidation of secondary amines using inorganic semiconductors has known little success.^4c–e^ Hence, it is highly desirable to develop new photocatalytic systems for the visible light-driven aerobic oxidation of secondary amines that can afford more synthetically useful dehydrated imines.

Owing to their interesting properties that may differ from those of the bulk counterparts, graphene-like semiconductor nanosheets with an ultrathin thickness (*e.g.*, ≤10 nm) are of great interest. Considerable efforts have been made to improve their synthesis, exploit their useful properties, and widen their application.^5^ Graphene-like nanosheets feature highly specific surface areas and interesting photo/electronic properties that may find significant application in areas ranging from electronics to catalysis.^6^ The “top-down” approach has been widely employed to fabricate semiconductor UTNSs by exfoliating the bulk materials^7^ in an appropriate solvent with the aid of suitable additives. The resulting materials have large lateral dimensions related to those of the parent bulk materials. In contrast, the “bottom-up” synthesis,^8^ which is based on reactions among different precursors, affords materials with considerably smaller dimensions. However, the preparation of semiconductor UTNSs *via* the “bottom-up” approach is difficult because a strong driving force is required for material growth in two dimensions, while a passive growth is noted in the third dimension. Colloidal synthesis is a powerful strategy to fabricate colloidal nanocrystals with controlled phases,^9^ morphology,^10^ and composition,^11^ thereby affording a potential route to preparing semiconductor nanosheets. Such controllable properties would have interesting application in energy storage and conversion,^5a,b^ photoelectrocatalytic water splitting,^7*b*^ photodetectors,^12^ and thermoelectric conversion.^7*a*^

Bismuth oxyhalides (BiOX, X = Cl, Br, I), a family of layered ternary oxide semiconductors, have recently been extensively studied in the photodegradation of organic pollutants^13^ in aqueous solution. BiOX displays a structure-dependent photocatalytic performance based on a layered assembly interleaved with [Bi_2_O_2_] slabs and double halogen atom slabs. The layered structure not only inhibits the recombination of photogenerated charge carriers (owing to internal electric fields formed between [Bi_2_O_2_] and the halogen layers), but also reduces the surface trapping of photogenerated carriers.^13*a*^ Such characteristics afford broader photocatalysis applications of BiOX materials beyond widely studied photodegradation of organic pollutants. BiOX materials are typically prepared using hydrothermal routes, which afford materials with hydrophilic surface properties and large thicknesses.^13d,14^ To date, only Xie *et al.* have reported the synthesis of BiOCl UTNSs using a hydrothermal process; the resulting UTNSs exhibit a thickness of 2.7 nm and excellent catalytic activity, related to the presence of defects, toward the photodecomposition of rhodamine B.^13*b*^

In the present study, we demonstrate the first successful colloidal synthesis of single-crystalline BiOCl UTNSs by the hydrolysis of BiCl_3_ in octadecylene (ODE) solution, assisted by *in situ* formation of water *via* reaction between oleylamine (OLA) and oleic acid (OA). The resulting BiOCl colloidal UTNSs (C-UTNSs) featured a well-defined morphology with areal dimensions of 30–45 nm and controllable thicknesses of 3.7–8 nm. Furthermore, BiOCl C-UTNSs exhibited remarkable light absorption ability in the visible light spectrum. Additionally, they exhibited hydrophobic surface properties, which is distinct from the hydrophilic feature of the widely studied BiOCl materials prepared by hydrothermal process. Subsequently, BiOCl C-UTNSs were investigated as visible light photocatalysts for the aerobic oxidation of secondary amines to corresponding imines in organic solvent at room temperature. BiOCl C-UTNSs displayed a high catalytic performance. In contrast, the hydrothermally synthesized BiOCl nanomaterials (nanoplates and UTNSs, designated as H-nanoplates and H-UTNSs, respectively) displayed poor-to-zero photocatalytic performance. Therefore, for the first time, we demonstrate the extension of the application of BiOCl materials to the photocatalytic synthesis of fine chemicals.

## Results and discussion

The synthesis of BiOCl C-UTNSs was performed in dry ODE solution in the presence of OLA, OA, and iron(iii) acetylacetonate (Fe(acac)_3_). The water required for the hydrolysis of BiCl_3_ was produced *in situ* upon reaction between OLA and OA; as such, the amount of water in the reaction system could be well controlled. Complete experimental details are provided in the ESI.†

**Fig. 1:**
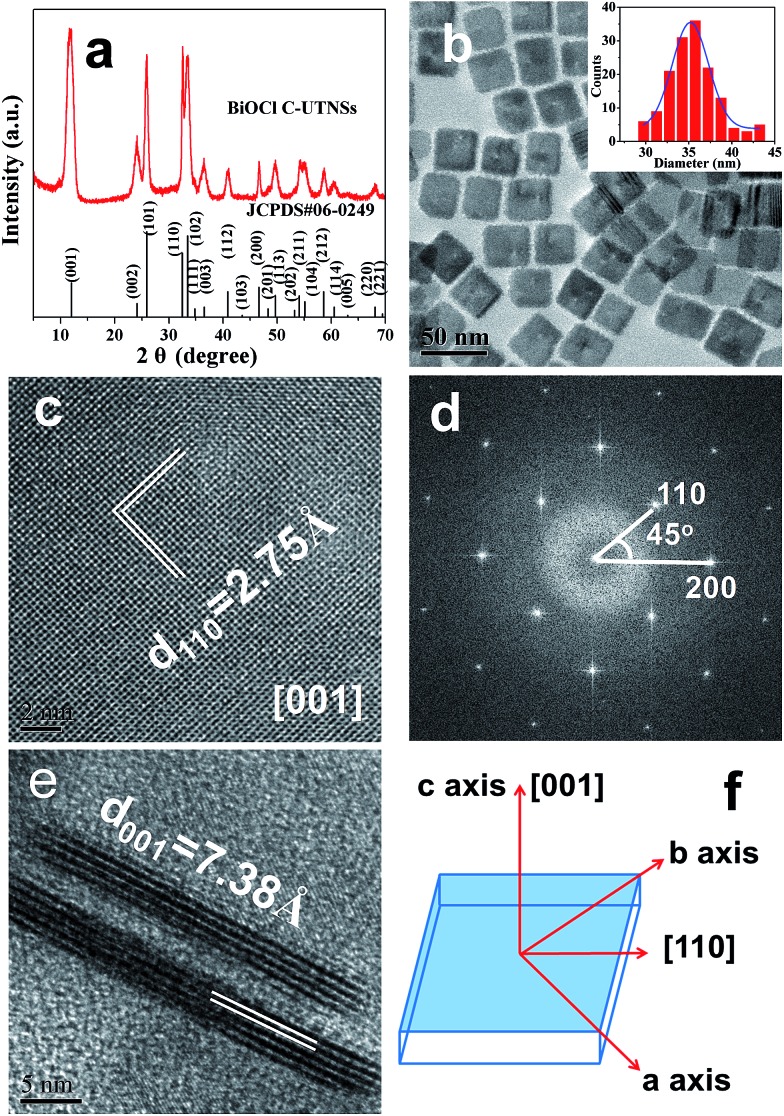
Structural characterization of BiOCl C-UTNSs: (a) XRD pattern; (b) TEM image and size distributions (inset); (c) plan-view HRTEM image and (d) corresponding FFT pattern; (e) lateral view HRTEM image; and (f) schematic illustration of the crystal orientation.

**Scheme 1:**
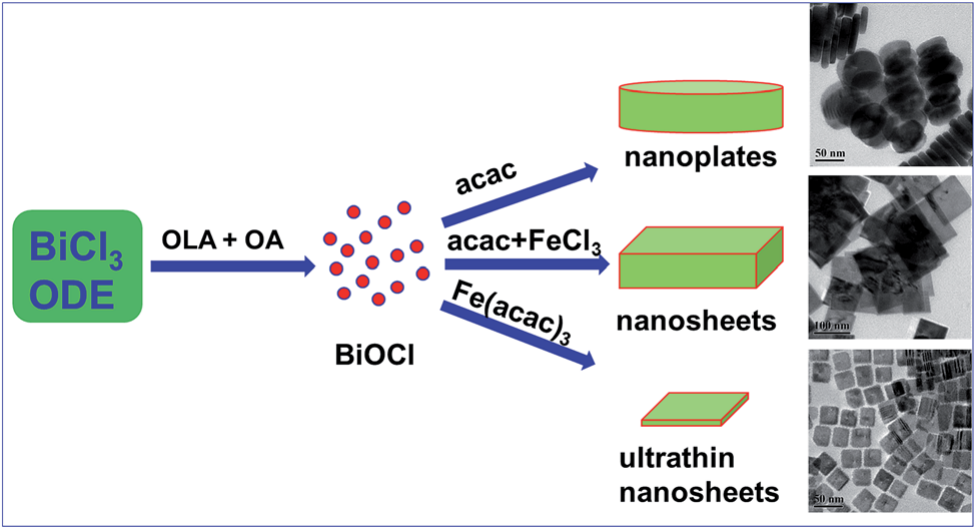
Schematic illustration for the proposed formation process of BiOCl materials by a facile colloidal approach.

Fig. 1a shows the powder X-ray diffraction (XRD) pattern of BiOCl C-UTNSs. All diffraction peaks could be indexed to the tetragonal phase of BiOCl with lattice parameters *a* = 0.3891 nm and *c* = 0.7369 nm (JCPDS no. 06-249), indicating the high purity of the product. Transmission electron microscopy (TEM) analysis (Fig. 1b) revealed that BiOCl C-UTNSs consisted of well-defined square nanosheets with areal dimensions of 30–45 nm (inset of Fig. 1b). High-resolution transmission electron microscopy (HRTEM) analysis (Fig. 1c) confirmed the highly crystalline characteristics of BiOCl C-UTNSs, and the corresponding fast Fourier transform (FFT) pattern (Fig. 1d) revealed the single-crystalline nature of the C-UTNSs. The lattice fringes with an interplanar lattice spacing (*d*) of 0.275 nm correspond to the (110) plane of tetragonal BiOCl. The angle between the adjacent spots labeled in the FFT pattern is 45°, which agrees with the theoretical value of the angle between the (110) and (200) planes of tetragonal BiOCl. Moreover, TEM analysis of the vertical nanosheets (Fig. 1e) showed that the thickness of BiOCl C-UTNSs was only ∼3.7 nm. Considering that *d*_001_ of BiOCl is 0.738 nm, it can be deduced that each BiOCl C-UTNS comprises approximately five [Cl–Bi–O–Bi–Cl] units. Based on the above results and the symmetries of tetragonal BiOCl (Fig. 1f), the bottom and top surfaces of the BiOCl UTNSs sample could be identified as {001} facets, whereas the four lateral surfaces could be identified as {110} facets. The percentage of {001} facets of BiOCl C-UTNSs was estimated by geometric calculation as 80%. It is widely accepted that the {001} facets of BiOCl possess a high oxygen density,^13a,15^ and the surface atoms can escape more easily from the lattice than from the inner atoms.^13*b*^ Consequently, the as-synthesized BiOCl C-UTNSs with exposed {001} facets are expected to exhibit more oxygen defects when compared with the hydrothermally synthesized BiOCl bulk materials. Interestingly, the thickness of the UTNSs could be tuned in the range of 3.7–8 nm by varying the reaction time (Fig. S1, ESI†), while the areal dimensions remained constant (Fig. S2, ESI†). The control experiments showed that the formation of well-defined BiOCl C-UTNSs was due to a synergistic effect between Fe^3+^ and acetylacetone (Fig. S3, ESI†), and the corresponding growth mechanisms for these BiOCl materials have been proposed in Scheme 1. Moreover, the well-defined morphology of BiOCl C-UTNSs could only be obtained when linear organic acids bearing more than eight carbons per molecule were used. In summary, the organic acids used in the synthesis significantly influence the formation of well-defined C-UTNSs (Fig. S4, ESI†).

**Fig. 2:**
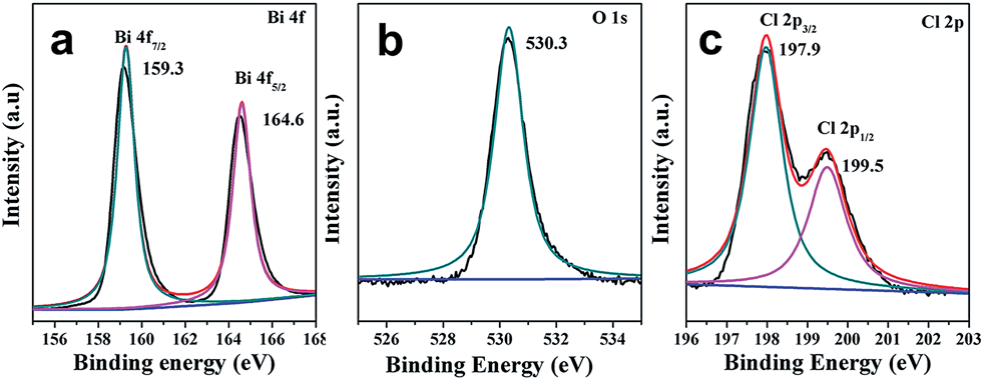
XPS spectra of BiOCl C-UTNSs: (a) Bi 4f; (b) O 1s; and (c) Cl 2p.

X-ray photoelectron spectroscopy (XPS) was further employed to characterize BiOCl C-UTNSs. As observed in Fig. 2a, the XPS spectra featured two intense peaks at 159.3 and 164.6 eV that were respectively assigned to Bi 4f_7/2_ and Bi 4f_5/2_ of Bi^3+^.^15,16^ The O 1s core level spectrum (Fig. 2b) was fitted with the peak at 530.3 eV, belonging to O^2–^ from a bismuth–oxygen bond in BiOCl. The absence of peaks at ∼531 eV was suggestive of the absence of surface OH^–^ or chemisorbed water on BiOCl C-UTNSs—hydrothermally synthesized BiOCl materials typically display a peak in this region.^17^ The peaks with binding energies of 197.9 and 199.5 eV (Fig. 2c), corresponding to Cl 2p_3/2_ and Cl 2p_1/2_, respectively, are characteristic of Cl^–^ in BiOCl. Quantitative XPS analysis of BiOCl UTNSs revealed Bi/O/Cl/Fe atomic ratios of ∼265 : 188 : 244 : 1. The oxygen content was lower than expected, indicative of the existence of a large concentration of oxygen vacancies in BiOCl C-UTNSs. The trace amounts of Fe were likely due to residual ions adsorbed onto the surface of the C-UTNSs during synthesis. Electron energy loss spectroscopy (EELS) analysis did not reveal any Fe signals, further confirming that the content of Fe was negligible in the prepared BiOCl C-UTNSs.

**Fig. 3:**
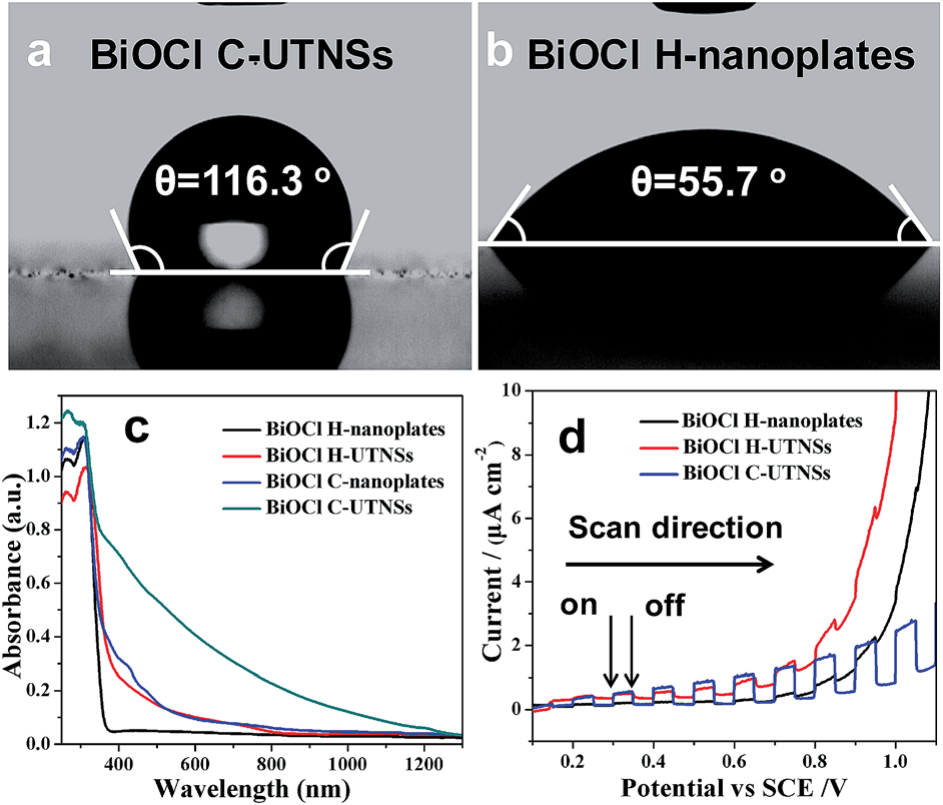
Surface water contact angle measurements of (a) BiOCl C-UTNSs and (b) H-nanoplates. (c) UV-Vis-NIR diffuse reflectance spectra and (d) transient photocurrent response of the different BiOCl materials.

Nanocrystals prepared by colloidal chemistry are capped by a layer of organic ligands and thus possess a hydrophobic surface. In contrast, materials prepared *via* a hydrothermal route show hydrophilic characteristics. For comparison purposes, BiOCl H-nanoplates and H-UTNSs were prepared using a hydrothermal route according to a reported procedure.^13*b*^ The corresponding TEM images and XRD patterns are shown in Fig. S5 and S6 in ESI,† respectively. The wettability of the corresponding BiOCl films was evaluated by surface water contact angle (CA) measurements. BiOCl C-UTNSs, a hydrophobic material, displayed a water CA of 116.3° (Fig. 3a). In contrast, BiOCl H-nanoplates displayed a lower water CA of 55.7° (Fig. 3b), indicative of the presence of a hydrophilic surface. Moreover, BiOCl H-UTNSs displayed a water CA of 0°, indicative of the superhydrophilic nature of the material (results are not shown because of the non-clarity of image of the surface water CA of this sample). Therefore, the BiOCl materials prepared by different approaches exhibit different surface properties.

The ultraviolet-visible-near infrared (UV-vis-NIR) diffuse reflectance spectra of BiOCl C-UTNSs and the hydrothermal materials were recorded to study their optical properties (Fig. 3c). Strong absorption in the visible light range was observed for BiOCl C-UTNSs that is typical for BiOCl materials with oxygen defects.^16^ Owing to the fact that BiOCl C-UTNSs were prepared in dry ODE solution under an Ar atmosphere and based on the XPS results, the visible light absorption is attributed to the presence of oxygen vacancies in BiOCl C-UTNSs. This hypothesis was confirmed by electron paramagnetic resonance (EPR) studies (Fig. S7, ESI†). BiOCl C-UTNSs showed an EPR signal at *g* = 2.001, which is typical of oxygen vacancies.^15,16^ In contrast, the control BiOCl H-nanoplates did not display any visible light absorption.^13d,14a,18^ The BiOCl C-UTNSs sample obtained in this study has abundant oxygen vacancies that lead to strong absorption in the visible light range. This observation was in stark contrast to that of BiOCl bulk materials that absorbed light in the ultraviolet region only owing to their relatively large band gap of ∼3.2 eV.

The photoelectrochemical properties of the BiOCl materials were assessed by measuring the transient photocurrents of the corresponding films on a fluorine-doped tin oxide substrate in a photoelectrochemical cell. All materials displayed positive photocurrents (anode current) (Fig. 3d), indicative of n-type semiconductor behaviors.^19^ The intensity of the photocurrent generated from the C-UTNSs was stronger (Fig. 3d) than that produced by the hydrothermally synthesized materials. These results were consistent with the UV-vis absorption results, whereby significantly increased absorption in the UV-vis range was observed for the materials that exhibited larger photocurrents. Additionally, the C-UTNSs were more stable and sensitive to illumination than the hydrothermally synthesized materials during the photoelectrochemical measurements (Fig. S8, ESI†). The above characteristics are important for exploiting the potential applications of such materials *e.g.*, photocatalytic synthesis of fine chemicals.

The combined strong absorption ability in the visible light region and sensitive response to light illumination makes BiOCl C-UTNSs excellent candidates for visible light-driven photocatalytic reactions. Furthermore, the oil-compatible characteristics of BiOCl C-UTNSs show potential for application in synthetic chemistry, thereby extending the application of such materials beyond photodegradation of pollutants in aqueous media. Hence, BiOCl C-UTNSs were examined as photocatalysts toward the photocatalytic aerobic oxidation of secondary amines into imines.

**Scheme 2:**
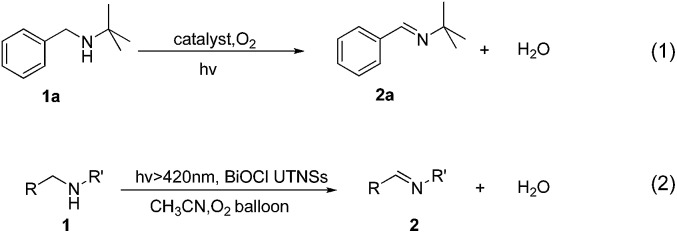
Photocatalytic reactions of different secondary amines to imines.

**Fig. 4:**
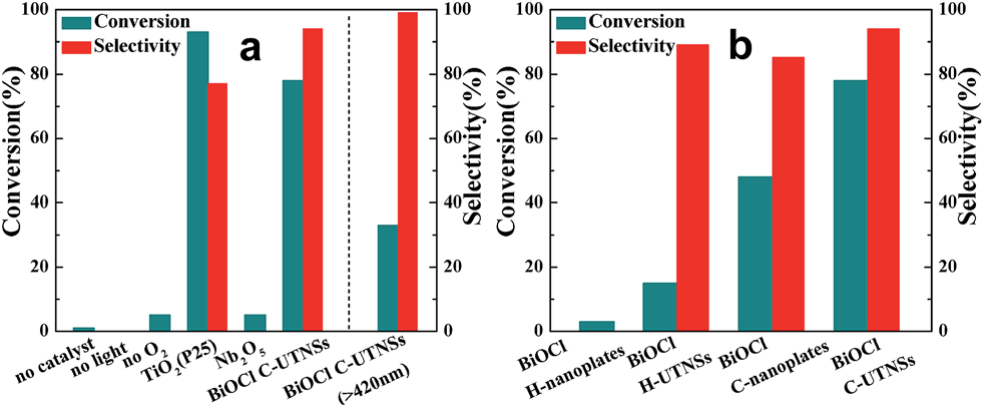
Photocatalytic aerobic oxidation of **1a** using different (a) semiconductor catalysts and (b) BiOCl materials. Reaction conditions: catalyst (0.077 mmol), substrate (0.1 mmol), CH_3_CN (4 mL), O_2_ atmosphere, 1 h, Xe lamp irradiation.

*N-t*-Butylbenzylamine (**1a**) was chosen as the model substrate (Scheme 2, Reaction (1)). The performances of the BiOCl materials and conventional photocatalytic semiconductors (Nb_2_O_5_ and TiO_2_ P25) were compared. The results showed that Nb_2_O_5_ displayed very low reactivity toward the photocatalytic aerobic oxidation of **1a** (Fig. 4a). The reaction improved in the presence of TiO_2_ (P25) that achieved a conversion of 93%, but a moderate selectivity of 77% in 1 h. In contrast, BiOCl C-UTNSs exhibited unique catalytic performance, with 78% conversion of **1a** and 94% selectivity for **2a** under irradiation with Xe lamp. Moreover, BiOCl C-UTNSs proved to be an effective visible light catalyst, achieving 33% conversion of **1a** and 99% selectivity for **2a** under light irradiation of *λ* > 420 nm in 1 h; nearly complete conversion with no loss in selectivity were obtained under prolonged reaction of 7 h.

To gain insights into the unique activity of BiOCl C-UTNSs toward the photooxidation of *N-t*-butylbenzylamine, control experiments were performed, including BiOCl materials prepared by different methods. The results are shown in Fig. 4b. As observed, BiOCl H-nanoplates displayed very poor activity (3% conversion) toward the photooxidation of **1a**. A slight improvement was obtained using H-UTNSs (15% conversion) in contrast to the remarkable improvement obtained in the presence of C-UTNTs (78% conversion). These results were consistent with the UV-vis absorption results (Fig. 3c). Materials that displayed significantly stronger visible light absorption possessed better photocatalytic activity toward Reaction (1) (Scheme 2). Thus, light absorption ability strongly influenced the catalytic activity of the BiOCl materials.

To further investigate the difference in the catalytic activities of the different BiOCl samples in this study, another hydrophobic BiOCl material (BiOCl C-nanoplates) was prepared by hydrolysis of BiOCl in the presence of acetylacetone and in the absence of Fe(acac)_3_ by colloidal synthesis. The TEM image of BiOCl C-nanoplates was presented in Fig. S3b,† and the corresponding surface water CA of 86° was obtained, just in the middle of the C-UTNSs and H-UTNSs (Fig. S9, ESI†). Both the BiOCl H-UTNSs and C-nanoplates showed comparable absorption spectra in the UV-vis range (Fig. 3c). However, BiOCl C-nanoplates showed a considerably higher photocatalytic activity (48% conversion) than BiOCl H-UTNSs (15% conversion) (Fig. 4b). Moreover, the reactivity of the BiOCl C-nanoplates is significantly inferior to that of C-UTNSs. These results suggest that the oil-compatible, hydrophobic characteristic of the BiOCl colloidal materials is an important factor to achieving high catalytic activities toward the photooxidation of *N-t*-butylbenzylamine.

To confirm the oxidative ability of BiOCl C-UTNSs under visible light irradiation at room temperature, different substituted *N-t*-butyl amines were investigated (Scheme 2, Reaction (2)). The high catalytic performance of BiOCl C-UTNSs could be extended to the oxidation of different substituted secondary amines. As presented in Table 1, variations in the substituent on the different positions of the phenyl moiety of the amine had little impact on the selectivity of the reaction (entries 1–8). Various substituted imines were obtained with high conversion (90–99%) and selectivity (89–99%). The oxidation of amines featuring electron-donating groups on the phenyl ring (Table 1, entries 2 and 3) proceeded more efficiently than that of amines with electron-withdrawing substituted groups (Table 1, entries 4–7). Regarding the oxidation of dibenzylamine, a considerable high selectivity (>99%) for the imine was obtained. These results show the superior performance of the prepared BiOCl C-UTNSs over different photocatalysts reported in the literature.^4c–e^

**Table 1:** Oxidation of secondary amines over BiOCl C-UTNSs under visible light illumination^*a*^

Entry	Substrate	Reaction time (h)	Conversion^*b*^ (%)	Selectivity^*b*^ (%)
1	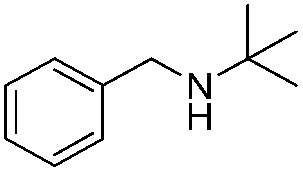	7	97	90
2	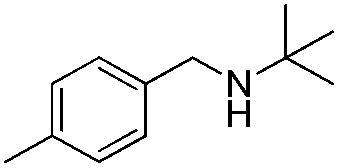	6.5	>99	95
3	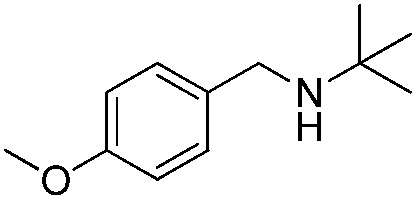	6	93	89
4	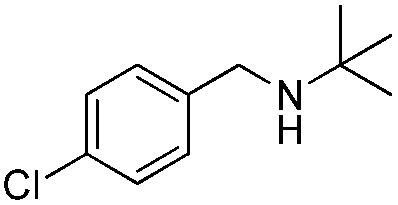	11	92	91
5	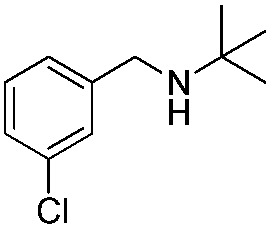	10	95	94
6	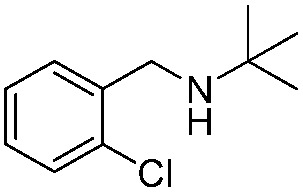	13	90	95
7	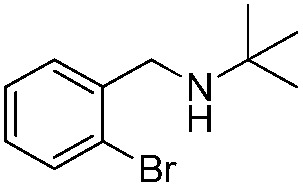	10	92	>99
8	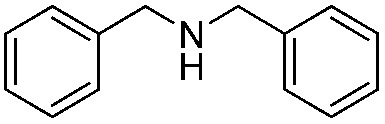	10	>99	>99

^*a*^Reaction conditions: *λ* > 420 nm, BiOCl C-UTNSs (20 mg), amine (0.1 mmol), CH_3_CN as a solvent (4 mL), oxygen balloon (1 atm), room temperature.

^*b*^Determined by gas chromatography using 1,4-diisopropylbenzene as the internal standard.

To demonstrate the practicality of the current reaction system, BiOCl C-UTNSs could be easily separated by a simple filtration and reused accordingly. No significant loss in activity or selectivity of the recovered catalyst was observed even after ten cycles (Fig. S10, ESI†). We have further performed XRD and TEM measurement to monitor the phase and morphology of the BiOCl C-UTNSs subjected to reactions (Fig. S11, ESI†). XRD patterns confirm that the phase of BiOCl was kept perfectly unchanged without any impurity detectable after the cycling reactions. TEM images revealed the square morphology was retained while a certain degree of agglomeration occurred after 4 cycles of reaction. These results clearly demonstrate that BiOCl C-UTNSs exhibit excellent stability and outstanding catalytic performance with high activity and selectivity toward the photocatalytic aerobic oxidation of secondary amines under visible light illumination. Therefore, the current study demonstrates a green strategy toward the photocatalytic aerobic oxidation of secondary amines to the corresponding imines using BiOCl C-UTNSs under visible light, as a driving force, at room temperature.

## Conclusions

A facile colloidal approach was developed to prepare well-defined single-crystalline BiOCl C-UTNSs. The UTNSs exhibited a square morphology with dimensions of 30–45 nm and thicknesses of 3.7–8 nm. They displayed strong absorption in the visible range that was attributed to the high content of oxygen vacancies in the nanosheets. BiOCl C-UTNSs featured high catalytic activity and excellent selectivity toward the aerobic oxidation of secondary amines to imines. The results suggested that both the light absorption ability in the visible range and hydrophobic surface property of BiOCl C-UTNSs afforded superior photocatalytic performances.
