# Supplementary material for: Well-defined BiOCl colloidal ultrathin nanosheets: synthesis, characterization, and application in photocatalytic aerobic oxidation of secondary amines
Source: Chem Sci. 2014 Dec 22;6(3):1873–8. doi: 10.1039/c4sc03229b (PMC5649566; doi:10.1039/c4sc03229b)
Supplement: Supplementary file 1 [file SC-006-C4SC03229B-s001.pdf]

Electronic Supplementary Material (ESI) for Chemical Science.
This journal is © The Royal Society of Chemistry 2014

Electronic Supplementary Information

Well-defined BiOCl Colloidal Ultrathin Nanosheets: Synthesis, Characterization, and Application in Photocatalytic Aerobic Oxidation of Secondary Amines †

Yihui Wu,‡ Bo Yuan,‡ Mingrun Li, Wen-Hua Zhang,* Yan Liu* and Can Li*

[a] State Key Laboratory of Catalysis, Dalian Institute of Chemical Physics, Chinese Academy of Sciences, and Dalian National Laboratory for Clean Energy, 457 Zhongshan Road, Dalian 116023, China

‡ These authors contributed equally to this work

e-mail: whzhang@dicp.ac.cn; yanliu503@dicp.ac.cn; canli@dicp.ac.cn

Experimental Section

Chemicals. Iron (III) acetylacetonate ($Fe(acac)_3$, ≥99.9%), oleylamine (OLA, 70%), oleic acid (OA, 90%) and PVP (Mw=55000) were purchased from sigma-Aldrich. octadecylene (ODE, 90%), $BiCl_3$ (99.997%), $FeCl_3$ (98%), acetylacetone (acac, 99%), formamide (99%), octanoic acid (OTA, 98+%), lauric acid (LA, 99.5%), behenic acid (BA, 85%), Bismuth(III) nitrate hydrate (99.999%), D-mannitol (99%), NaCl (99.99%), N-t-butylbenzylamine(96%), CH_3CN(>99.5%), 1,4-Diisopropylbenzene(99%) were obtained from Alfa Aesar. Na_2SO_4, n-hexane and isopropanol, TiO_2 (Degussa P25), Nb_2O_5(>99.5%) were obtained from sinopharm chemical reagent Co., Ltd. OLA, OA and ODE were dried under vacuum at 100 ^{o}C for 5 h before use.

Synthesis of BiOCl C-UTNSs by colloidal approach. Typically, 106 mg (0.3 mmol) of $Fe(acac)_3$ and 95 mg (0.3 mmol) of $BiCl_3$, 400 μL of OLA, 400 μL of OA, and 4.5 mL of ODE were first loaded into a flask of 50 mL capacity. The flask was degassed by a vacuum pump for 30 min to remove low-boiling point impurities at 100 ^{o}C and then backfilled with argon. Afterwards, the temperature wa increased to 160 ^{o}C and maintained at this temperature for 30 min to finish the reaction. Subsequently, the solution was cooled down to 60 ^{o}C, then precipitated with 10 mL of isopropanol and centrifuged at 8000 rpm for 5 min. The upper solution was discarded and n-hexane was added to disperse the nanocrystals. Then the product was further purified by adding a certain amount of isopropanol and centrifuging. This process was repeated for three times to yield the nanocrystal product that could be dispersed in common organic solvents such as toluene for later use.

Synthesis of BiOCl C-nanoplates by colloidal approach. Typically, 95 mg (0.3 mmol) of $BiCl_3$, 400 μL of OLA, 400 μL of OA, and 4.5 mL of ODE were first loaded into a flask of 50 mL capacity. The flask was degassed by a vacuum pump for 30 min to remove low-boiling point impurities at 100 ^{o}C and then backfilled with argon. 200 μL of acetylacetone (acac) was injected, and then the temperature was increased to 160 ^{o}C and maintained at this temperature for 30 min to finish the reaction. Subsequently, the solution was cooled down to 60 ^{o}C, then precipitated with 10 mL of isopropanol and centrifuged at 8000 rpm for 5 min. The upper solution was discarded and n-hexane was added to disperse the nanocrystals. Then the product was further purified by adding a certain amount of isopropanol and cen-trifuging. This process was repeated for three times to yield the nanocrystal product that could be dis-persed in common organic solvents such as toluene for later use.

Synthesis of BiOCl H-UTNSs by hydrothermal approach. BiOCl H-UTNSs were prepared according to the reported process via hydrothermal method[1]. In a typical synthesis, 0.486 g of $Bi(NO_3)_3 \cdot xH_2O$ and 0.400 g PVP were dissolved in 25 mL of 0.1 M mannitol solution with vigorous stirring for 10 min.

Then, 5 mL of saturated NaCl solution was added dropwise into the above mixture, yielding a uniform white suspension. After another 10 min of agitation, the mixture was transferred into a Teflon-lined stainless steel autoclave of 50 mL capacity, which was heated at a temperature of 160 °C for 3 h and then cooled to room temperature naturally. The resulting solid powder was collected by centrifugation and washed with deionized water three times to remove residual ions. The final products were then dried at 60 °C for 4 h for further characterization.

Synthesis of BiOCl H-nanoplates by hydrothermal approach. BiOCl hydrothermal nanoplates were prepared according to the reported process via hydrothermal method[1]. In a typical synthesis, 0.486 g of $Bi(NO_3)_3 \cdot xH_2O$ was dissolved in 25 mL of 0.1 M mannitol solution with vigorous stirring for 10 min. Then, 5 mL of saturated NaCl solution was added dropwise into the above mixture, yielding a uniform white suspension. After another 10 min of agitation, the mixture was transferred into a Teflon-lined stainless steel autoclave of 50 mL capacity, which was heated at a temperature of 160 °C for 3 h and then cooled to room temperature naturally. The resulting solid powder was collected by centrifugation and washed with deionized water three times to remove residual ions. The final products were then dried at 60 °C for 4 h for further characterization.

Physical Characterization. X-ray diffraction (XRD) analyses were performed on a Rigaku RINT D/Max- 2500 powder diffraction system using Cu Kα radiation source (λ = 1.541 Å) operating at 40 kV and 200 mA with a scaning rate of 5°/ min in the 2θ range of 5-70° at a step size of 0.02 s. The TEM images showing the morphology of the NCs were obtained on a FEI TECNAI G^2 spirit microscope, operating at an accelerating voltage of 100 kV. HRTEM images and fast Fourier transform (FFT) patterns were obtained with a FEI TECNAI F30 S-Twin (FEI company) with an accelerating voltage of 300 kV. UV-vis-NIR diffuse-reflectance spectroscopy (DRS) spectra of BiOCl were acquired from a Cary 5000 spectrophotometer. Electron paramagnetic resonance (EPR) spectra were recorded with a Brucker EPR A200 spectrometer with a microwave frequency of 9.41 GHz. X-ray photoelectron spectroscopy (XPS) were recorded on a Thermo ESCALAB 250Xi spectrometer with a Al Kα (hυ=1486.6 eV) radiation source, using C 1s (285.0 eV) as reference. Transient photocurrent measurements were conducted in a quartz cell in aqueous 0.5 M Na_2SO_4 using 300 W Xe lamp illumination with a Saturated Calomel Electrode (SCE) as reference electrode and a Pt foil as counter electrode. The photoelectrochemical response of the ultrathin BiOCl ultrathin nanosheets films, which sprayed onto FTO, was assessed with a bias potential of 0.6 V under 20 s on/off chopped illumination. The surface water contact angle were measured on a Angle Contact Metering System (SL200B).

Catalytic characterizaiton. Typically, the photocatalytic amine oxidation was carried out under irradiation by a 300 W Xe lamp (with cut-off light below 420 nm if necessary) with stirring bar in a 15 mL

Pyrex reaction cell. The Pyrex glass bottle was sealed with a glass stopper, connected to an O_2 balloon and surrounded by cool water between 18 ^{o}C and 22 ^{o}C. The substrate (0.1 mmol) in the solvent of CH_3CN (4 mL) and BiOCl (20 mg) was introduced into the reactor. The suspension was vigorous stirred and irradiated from the side face with a cold water mirror in front. After the reaction, 300 μL of the suspension was extracted and the catalyst was removed by centrifugation. Then, the mixture were analyzed by GC using 1,4-diisopropylbenzene as the internal standard.

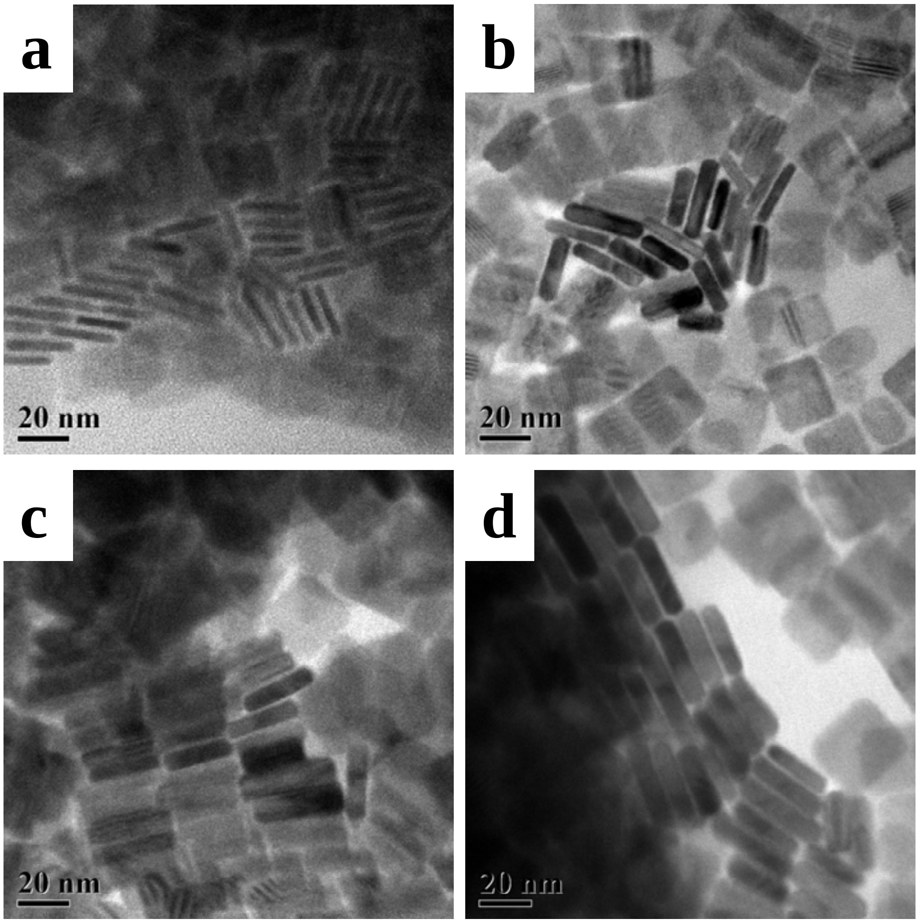

Fig. S1 Lateral-direction viewed TEM images of BiOCl UTNSs obtained with different reaction times: a) 30 min (~3.7 nm), b) 60 min (~5.5 nm), c) 180 min (~7 nm), and d) 300 min (~8 nm)

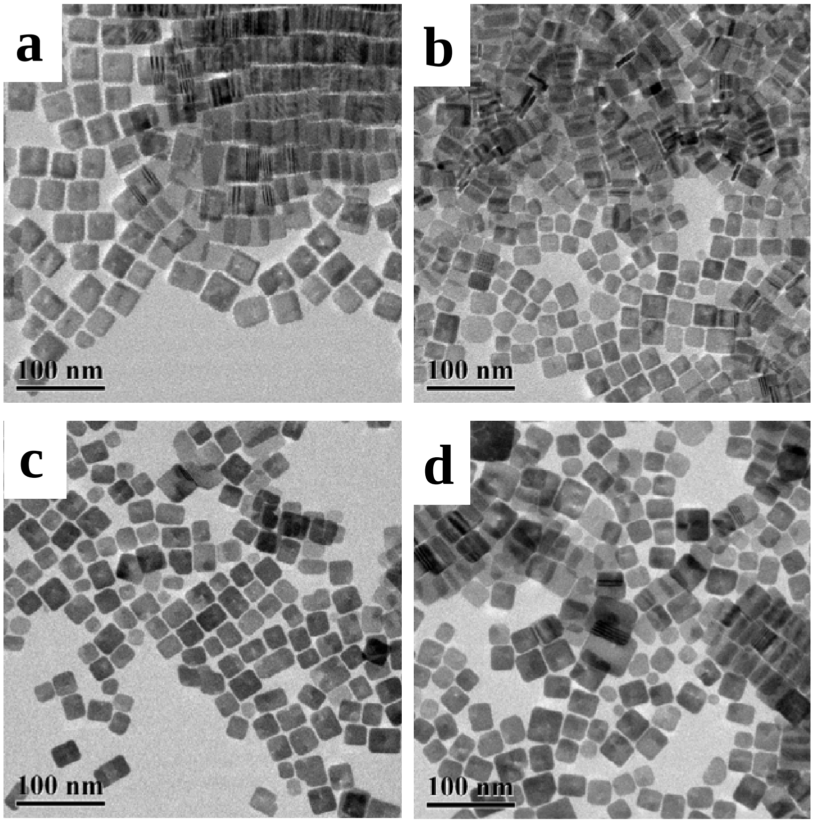

Fig. S2 Plane-viewed TEM images of BiOCl UTNSs obtained with different reaction times: a) 30 min, b) 60 min, c) 180 min, and d) 300 min.

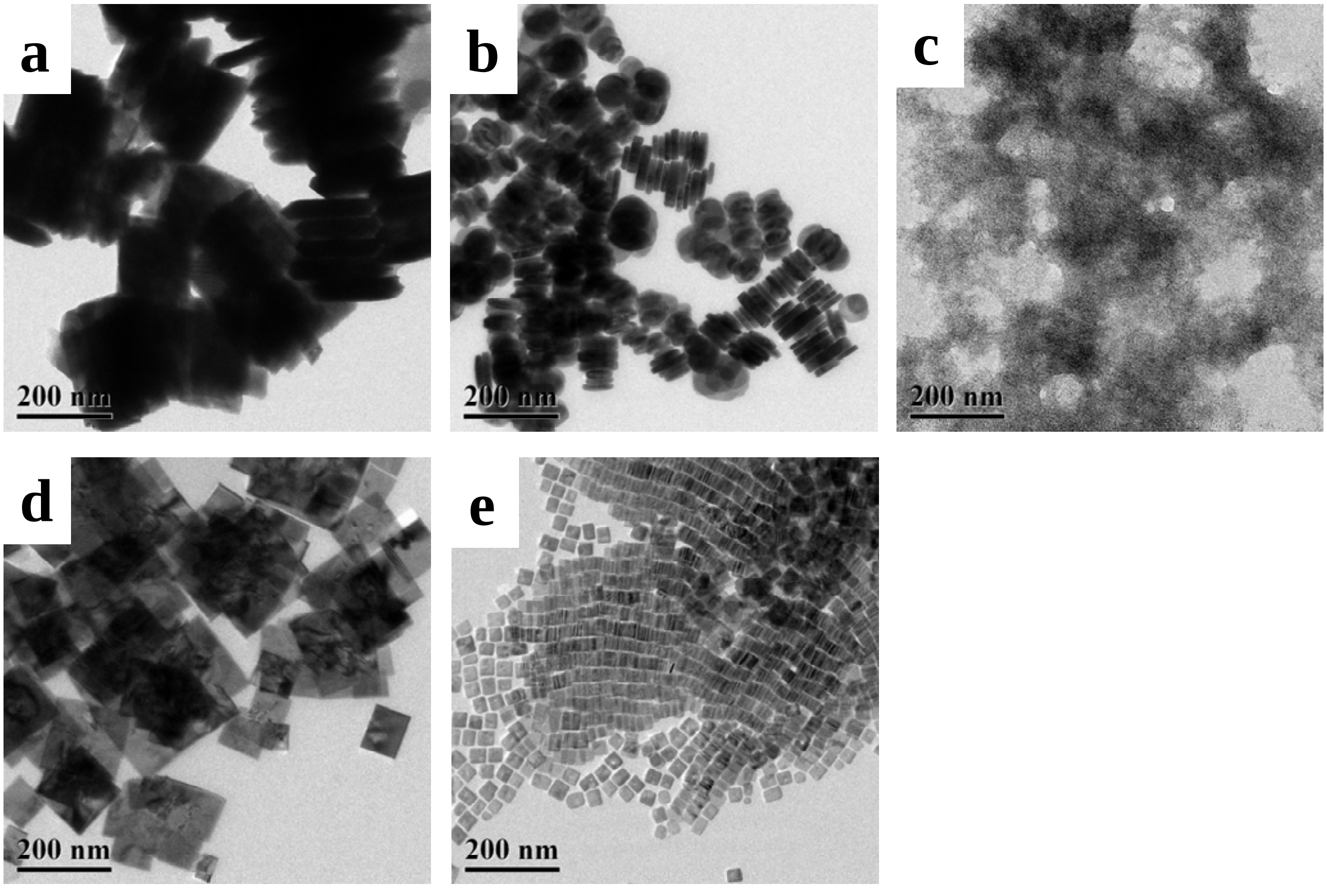

Fig. S3 TEM images of BiOCl obtained by adding different materials into the reaction mixture: a) without metal ions and acac, b) acac, c) $FeCl_3$, d) $FeCl_3$ and acac, e) $Fe(acac)_3$.

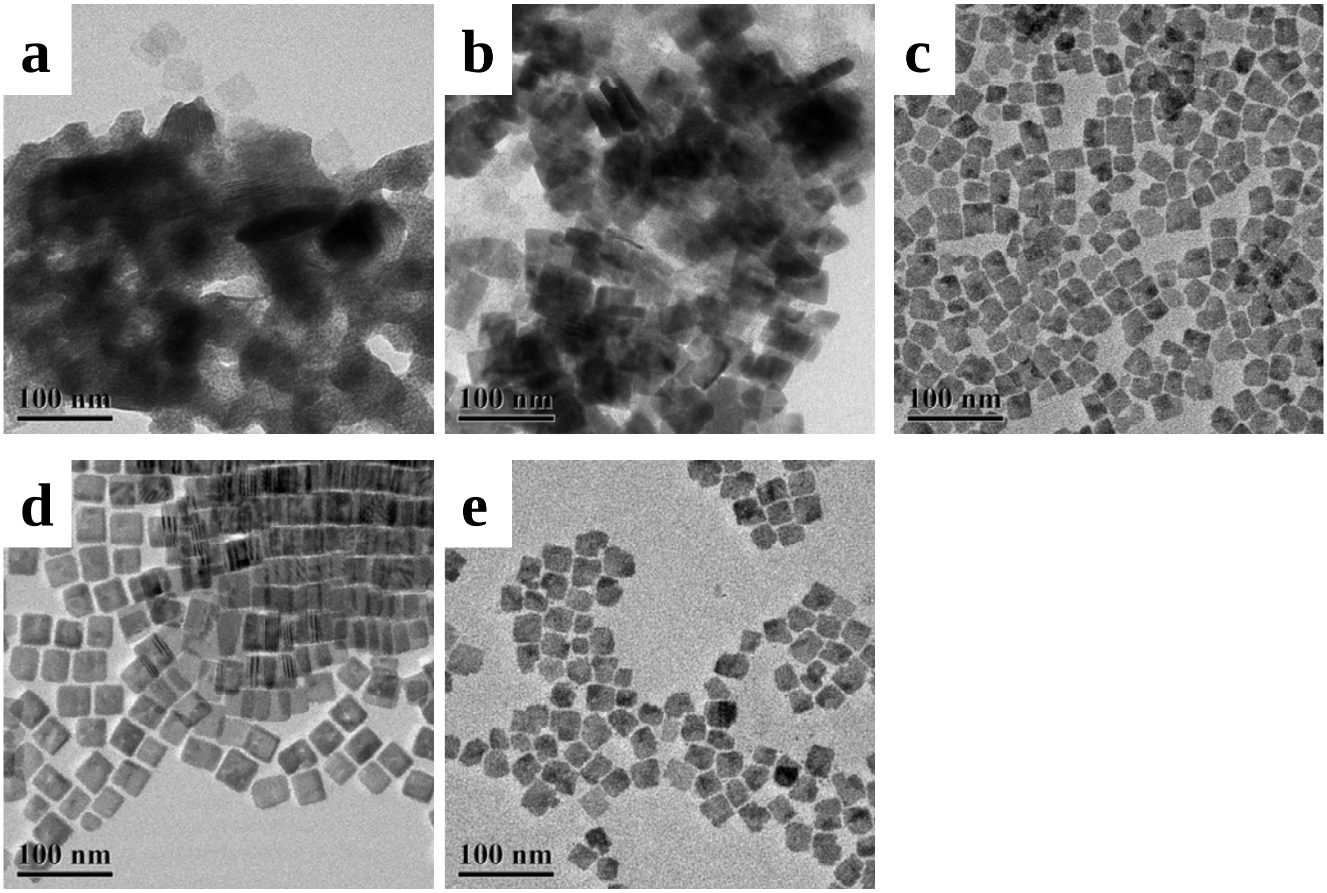

Fig. S4 TEM images of BiOCl obtained by adding different organic acids: a) without organic acid, b) octanoic acid, c) lauric acid, d) oleic acid, and e) behenic acid.

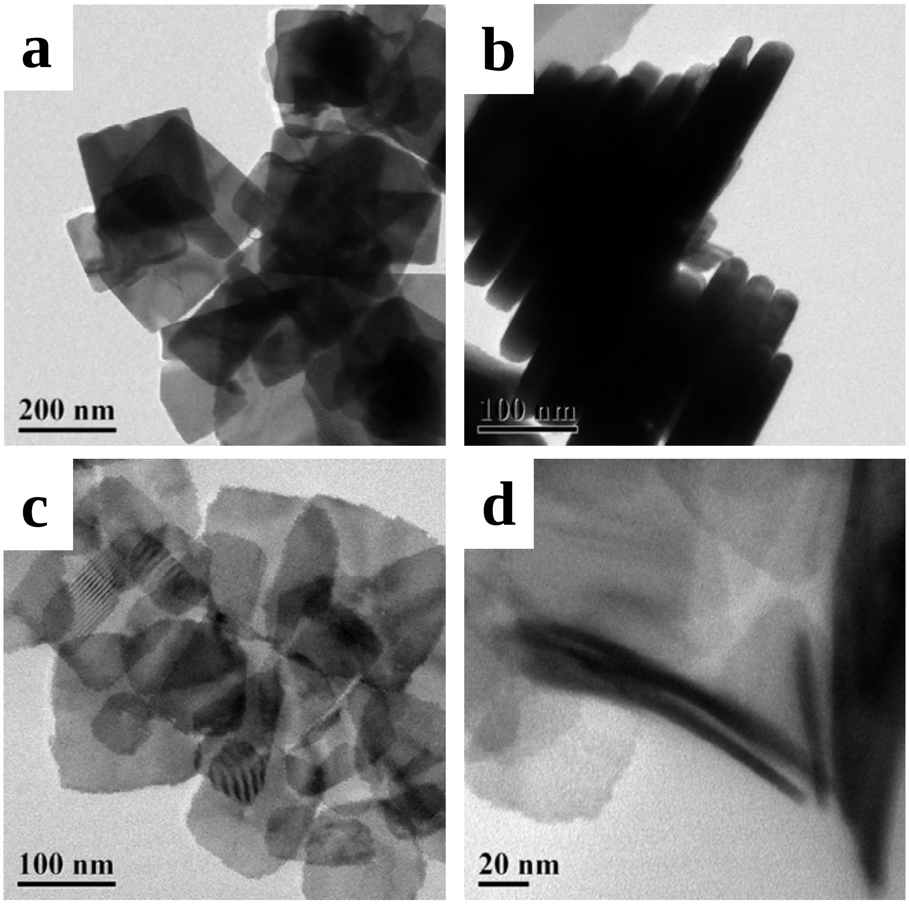

Fig. S5 (a) plane-viewed TEM images of the BiOCl H-nanoplates, (b) lateral-direction viewed TEM image of BiOCl H-nanoplates, **(c)** plane-viewed TEM images of the BiOCl H-UTNSs, (d) lateral-direction viewed TEM image of BiOCl H-UTNSs,

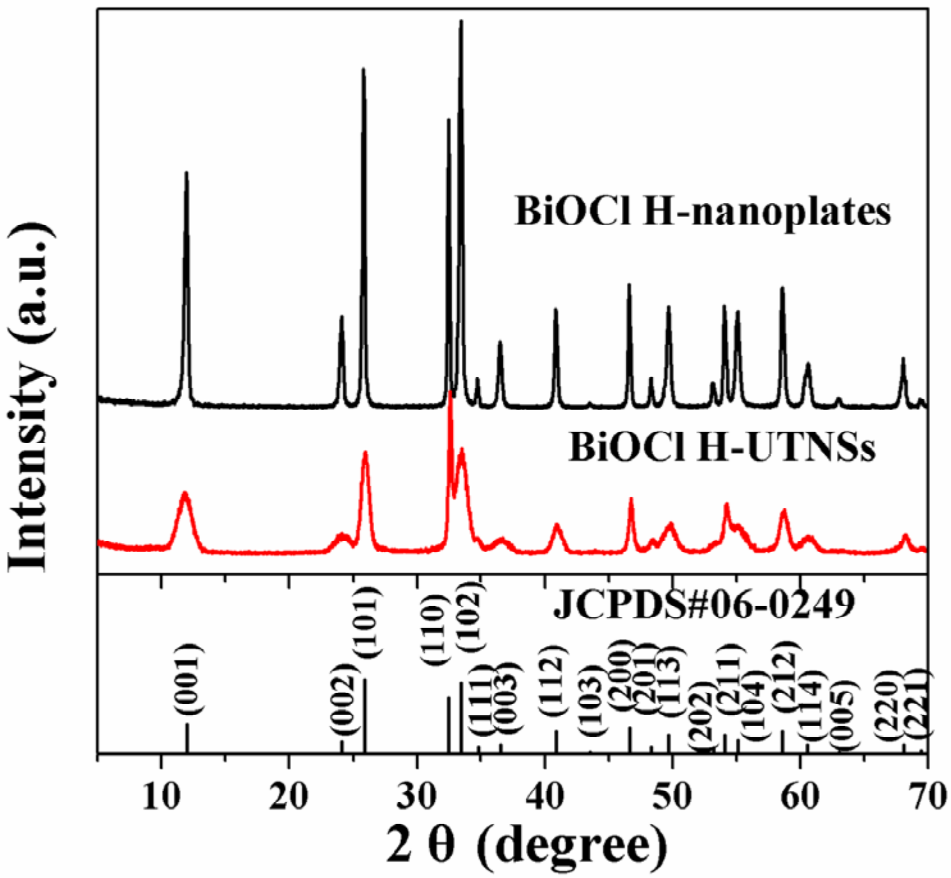

Fig. S6 XRD patterns of BiOCl hydrothermal materials.

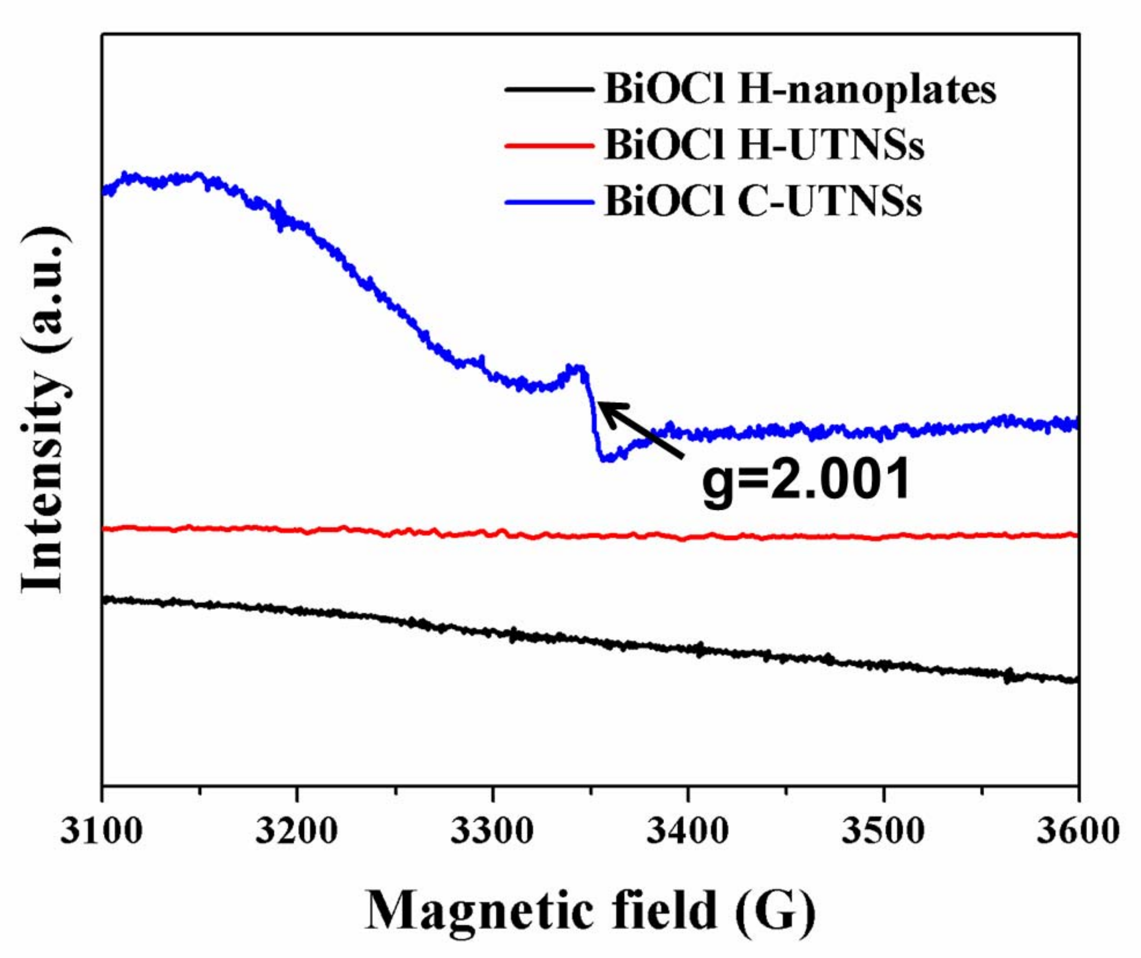

Fig. S7 EPR spectrum of the BiOCl H-nanoplates, BiOCl H-UTNSs and BiOCl C-UTNSs.

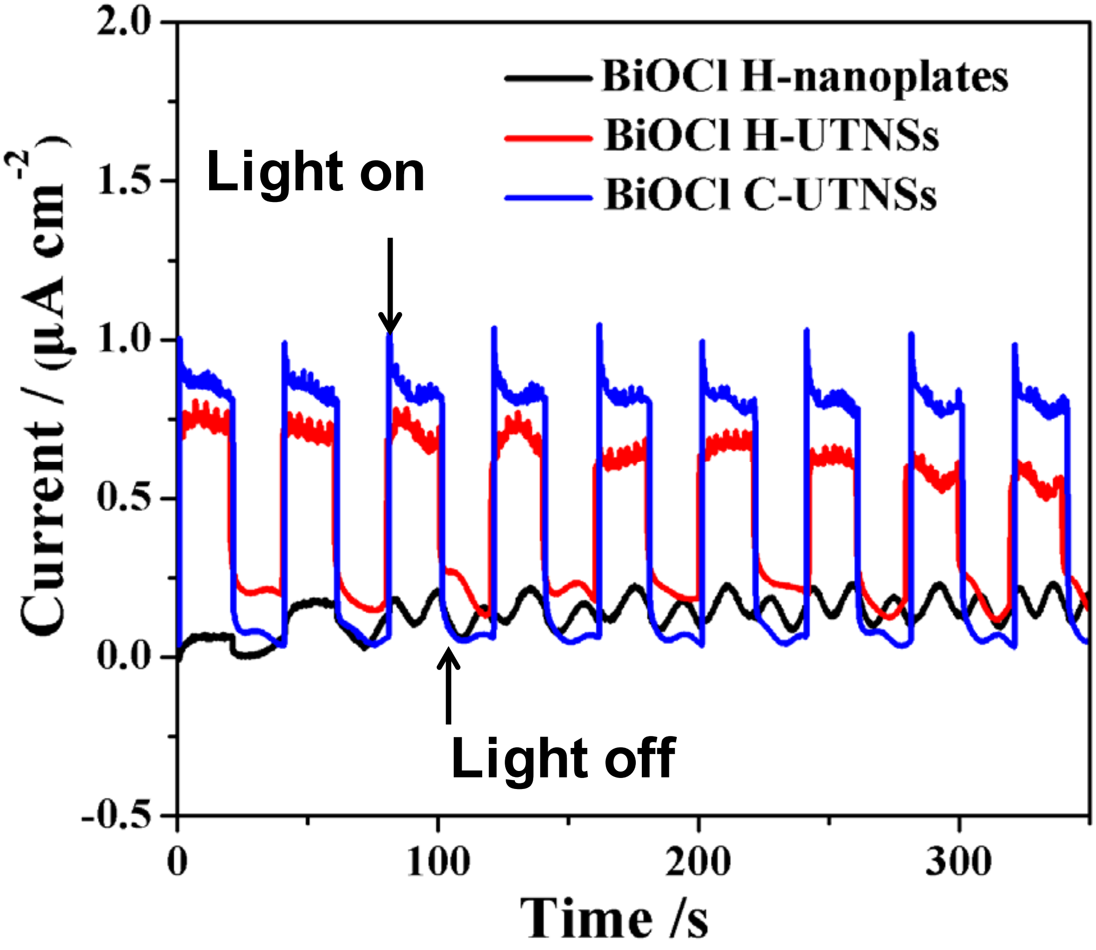

Fig. S8 Transient photocurrent response of BiOCl materials under a constant bias of 0.6 V.

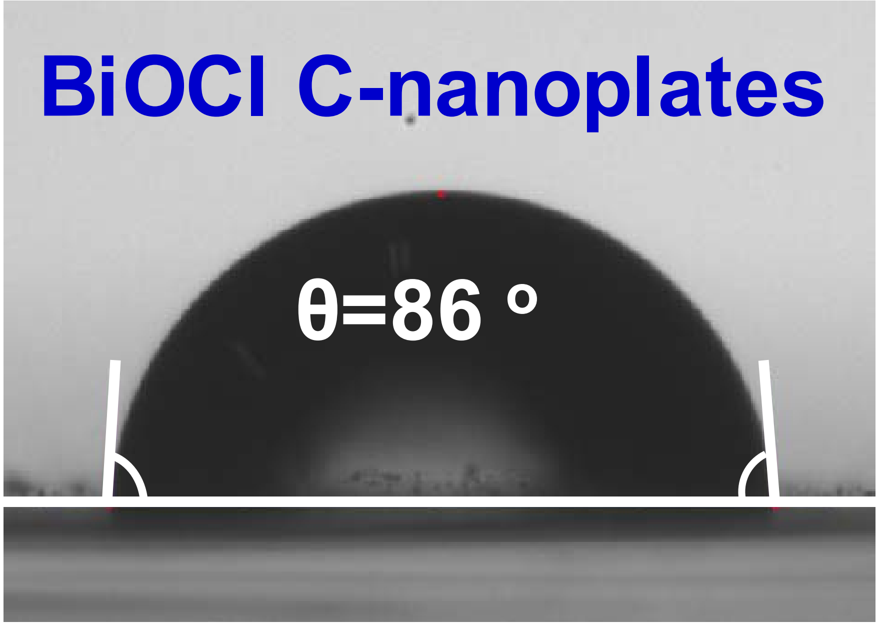

Fig. S9 The surface water contact angle of BiOCl C-nanoplates.

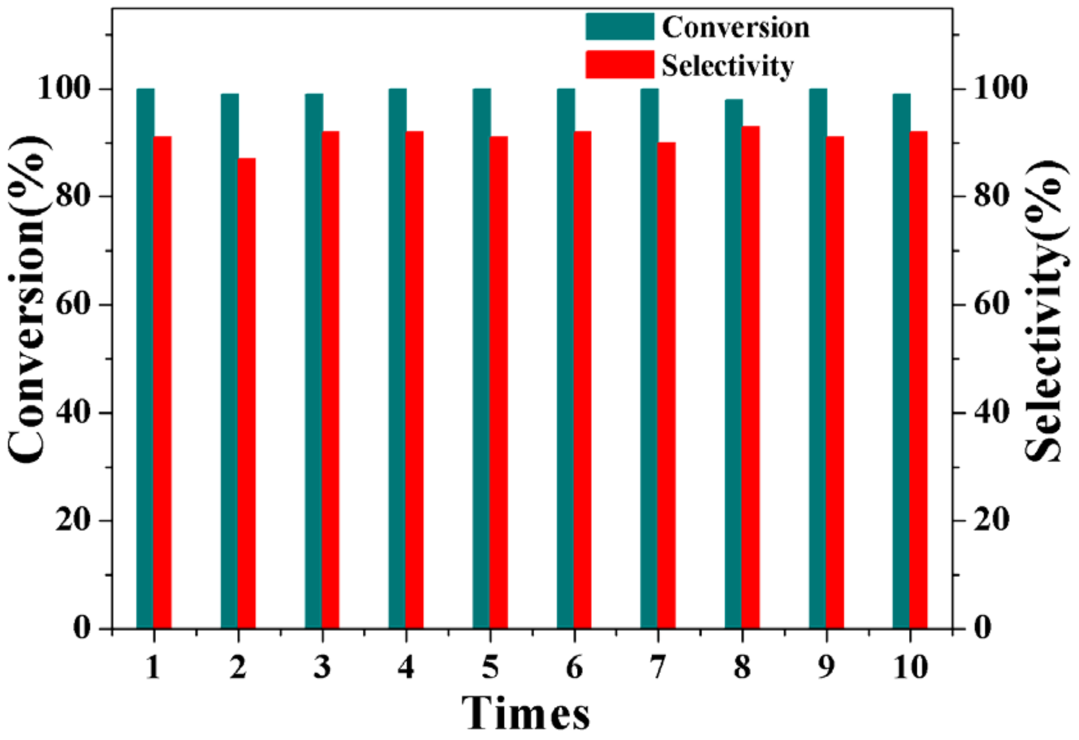

Fig. S10 The recycle test on the photocatalytic oxidation of N-t-butylbenzylamine.

Reaction conditions: λ>420 nm, 0.077 mmol of catalyst, 0.1 mmol of substrate, 4 mL of CH_3CN, O_2 balloon, and reaction was lasted for 7 hour.

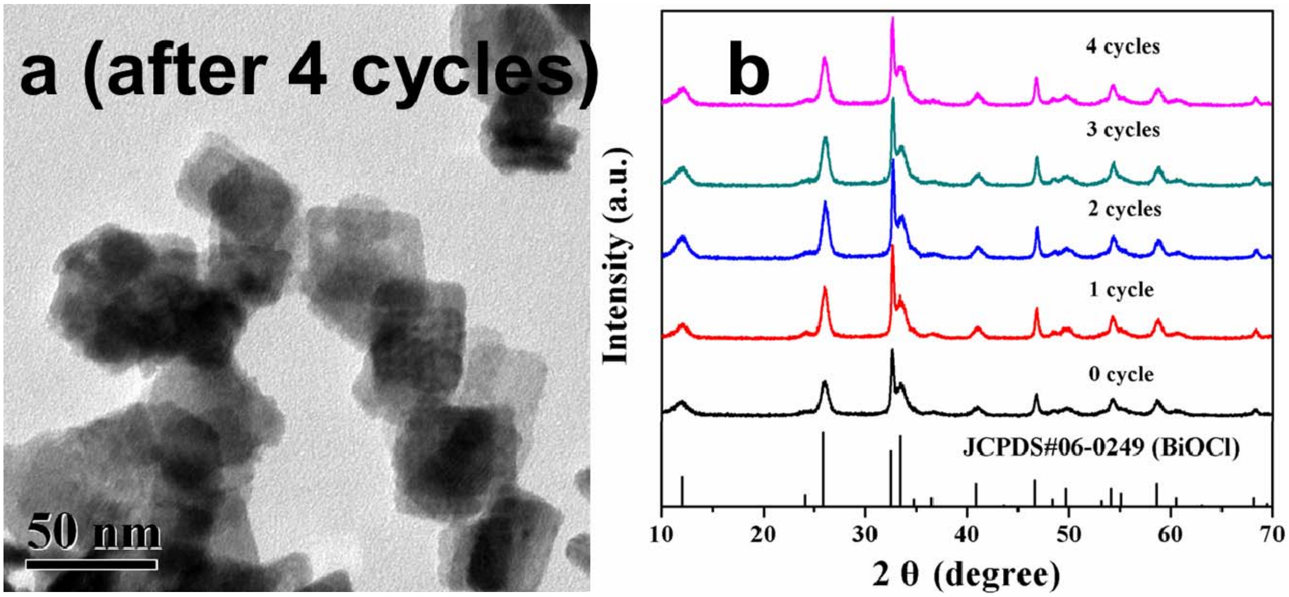

Fig. S11 TEM image (a) and XRD patterns (b) of BiOCl C-UTNSs after different reaction cycles.

REERENCES.

1. Guan, M.; Xiao, C.; Zhang, J.; Fan, S.; An, R.; Cheng, Q.; Xie, J.; Zhou, M.; Ye, B.; Xie, Y. *Journal of the American Chemical Society* **2013**, *135*, 10411-10417.
